# Supplementary material for: Engineered disorder in CO2 photocatalysis
Source: Nat Commun. 2022 Nov 23;13:7205. doi: 10.1038/s41467-022-34798-1 (PMC9684568; doi:10.1038/s41467-022-34798-1)
Supplement: Supplementary file 1 — Supplementary Information [file 41467_2022_34798_MOESM1_ESM.docx]

**Supplementary Information**

**Engineered Disorder in CO_2_ Photocatalysis**

*Zhao Li ^1,2,3,10^, Chengliang Mao ^2,10^, Qijun Pei ^4^, Paul N. Duchesne ^5^, Teng He ^4^, Meikun Xia ^2^, Jintao Wang ^4^, Lu Wang ^6^, Rui Song ^1,2,3^,  Feysal M. Ali^2^, Débora Motta Meira ^7,8^, Qingjie Ge ^4^, Kulbir Kaur Ghuman ^9*^, Le He ^1,3^, Xiaohong Zhang ^1,3*^, Geoffrey A. Ozin ^2*^*

*^1^ Institute of Functional Nano & Soft Materials (FUNSOM), Soochow University, 199 Ren'ai Road, Suzhou, 215123, Jiangsu, PR China*

*^2^ Solar Fuels Group, Department of Chemistry, University of Toronto, 80 St. George Street, Toronto, Ontario M5S 3H6, Canada*

*^3^ Jiangsu Key Laboratory of Advanced Negative Carbon Technologies, Soochow University, Suzhou, 215123, Jiangsu, PR China*

*^4^ Dalian Institute of Chemical Physics, Chinese Academy of Sciences, Dalian 116023, Liaoning, China*

*^5^ Department of Chemistry, Queen’s University, 90 Bader Lane, Kingston, ON K7L 3N6, Canada*

*^6^ The Chinese University of Hong Kong, Shenzhen, 518172 Shenzhen, Guangdong, People’s Republic of China.*

*^7^ CLS@APS, Advanced Photon Source, Argonne National Laboratory, Lemont, IL, 60439, USA.*

*^8^ Canadian Light Source Inc., 44 Innovation Boulevard, Saskatoon, SK S7N 2V3, Canada*

*^9^ Institut National de la Recherche Scientifique, Centre Énergie, Matériaux et Télécommunications, 1650 Boul. Lionel Boulet, Varennes, QC J3X 1S2, Canada*

*^10^ These authors contributed equally to this work.*


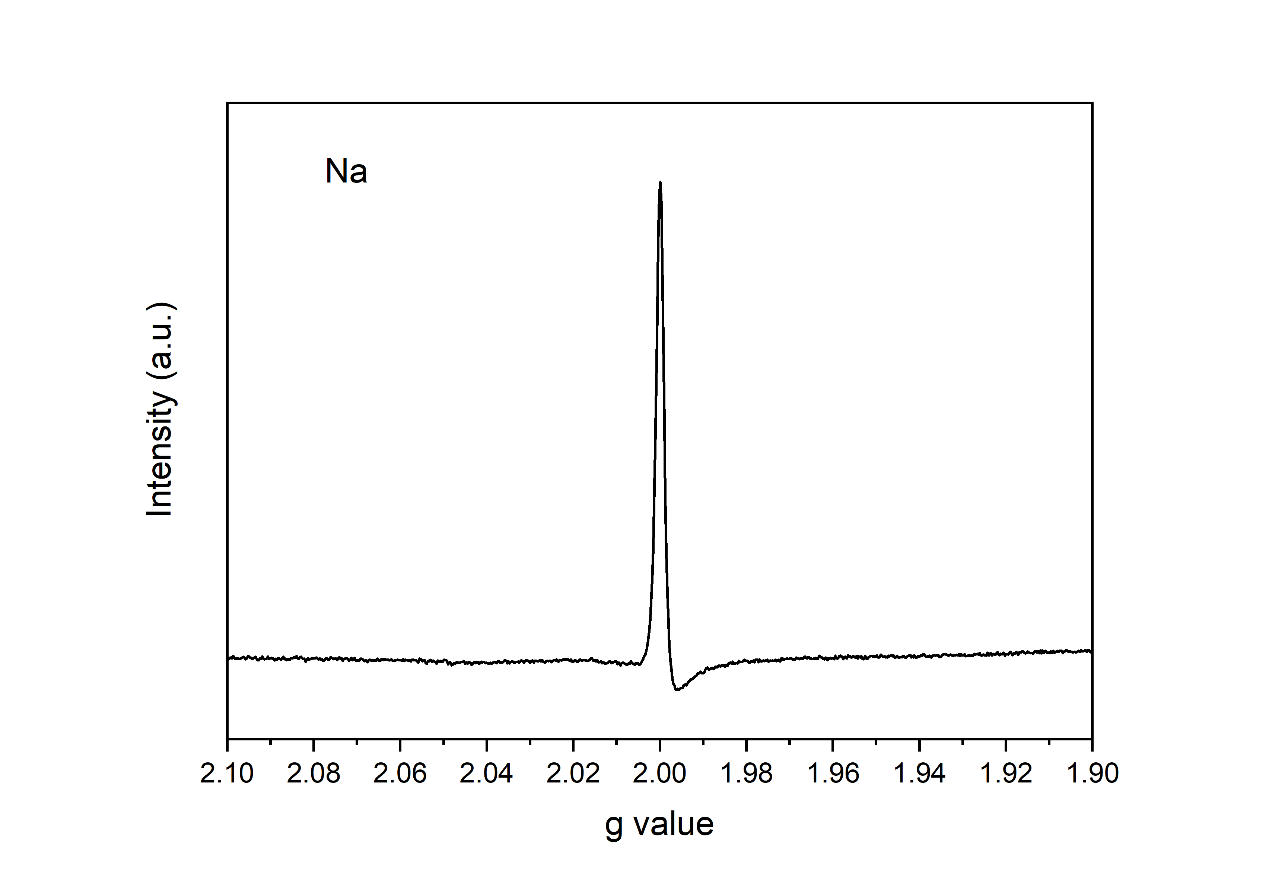


**Supplementary Fig. 1 |** EPR spectra of Na showing a Dysonian line shape.


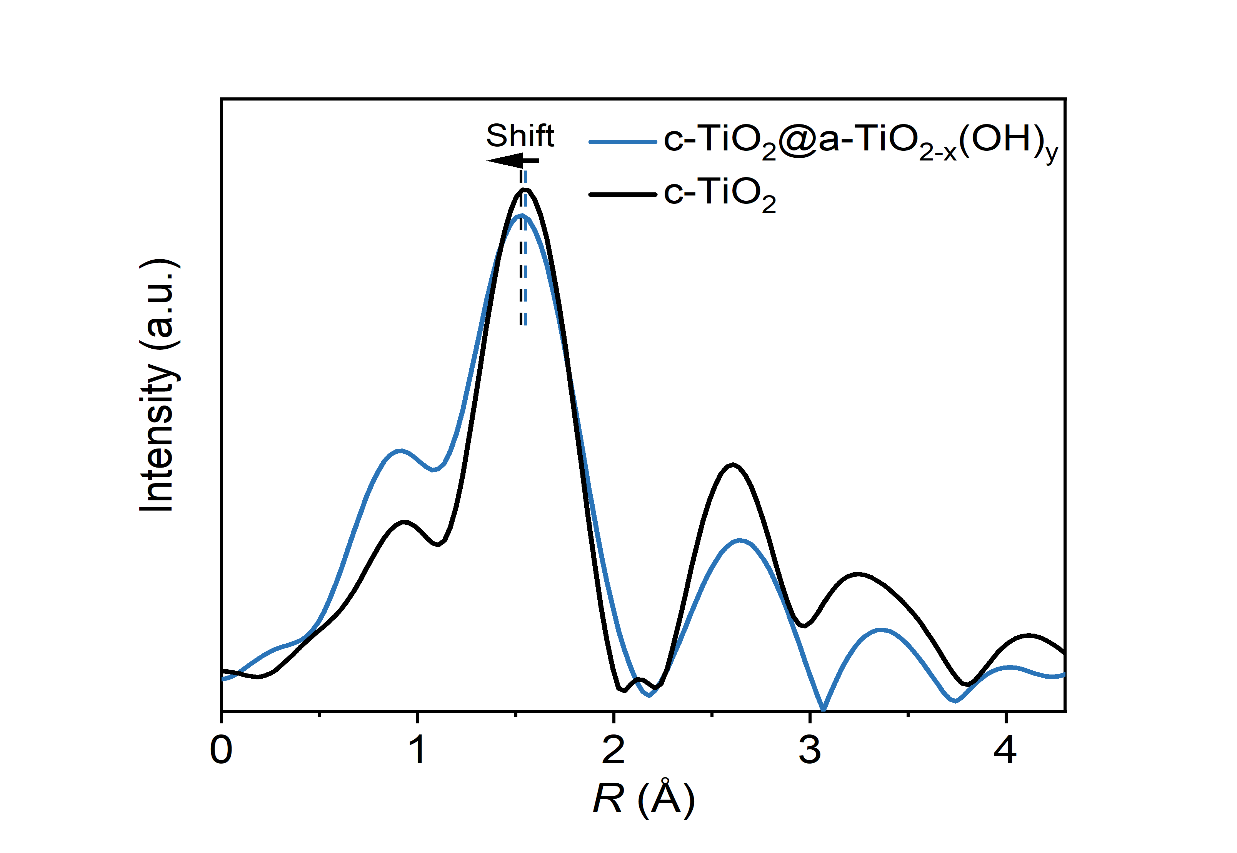


**Supplementary Fig. 2 |** Fourier-transformed Ti K-edge EXAFS spectra for c-TiO_2_ and c-TiO_2_@a-TiO_2-x_(OH)_y_.


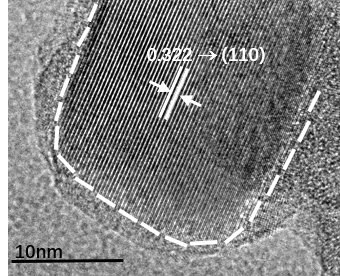


**Supplementary Fig. 3 |** HR-TEM micrograph of c-TiO_2_@a-TiO_2-x_(OH)_y_ (Rutile phase), the amorphous/crystalline interface is marked with dotted line. A distance of 0.322 nm is in accordance with the (110) planes in the rutile phase.

**Supplementary Fig. 4 |** HR-TEM micrograph of c-TiO_2_ (commercial P25).


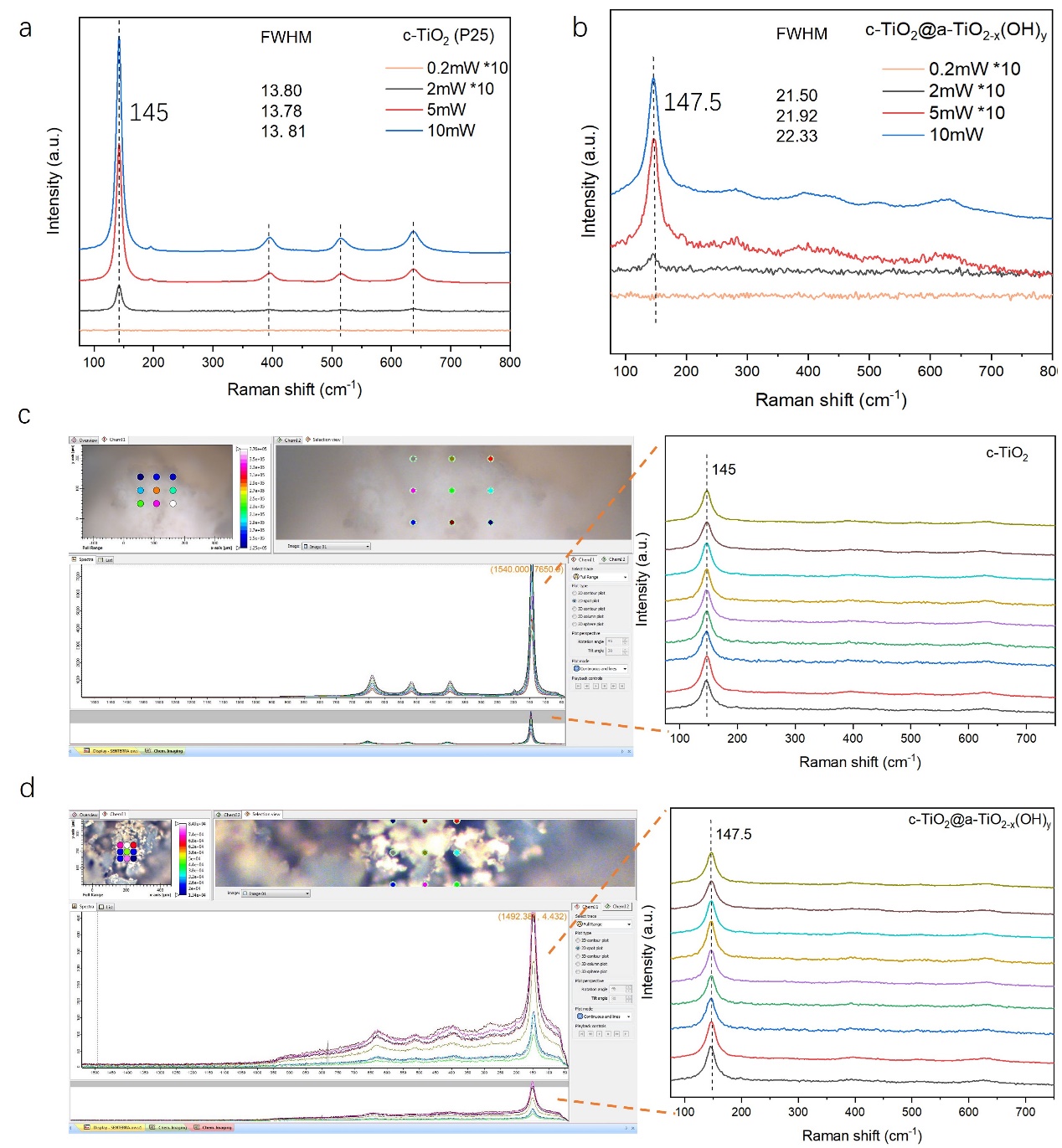


**Supplementary Fig. 5 |** Laser power-dependent Raman spectra of c-TiO_2_ (**a**) and c-TiO_2_@a-TiO_2-x_(OH)_y_ (**b**) under different intensity of laser power, and corresponding 3*3 Raman mapping (**c, d**). *10 represents multiplying the intensity by 10.


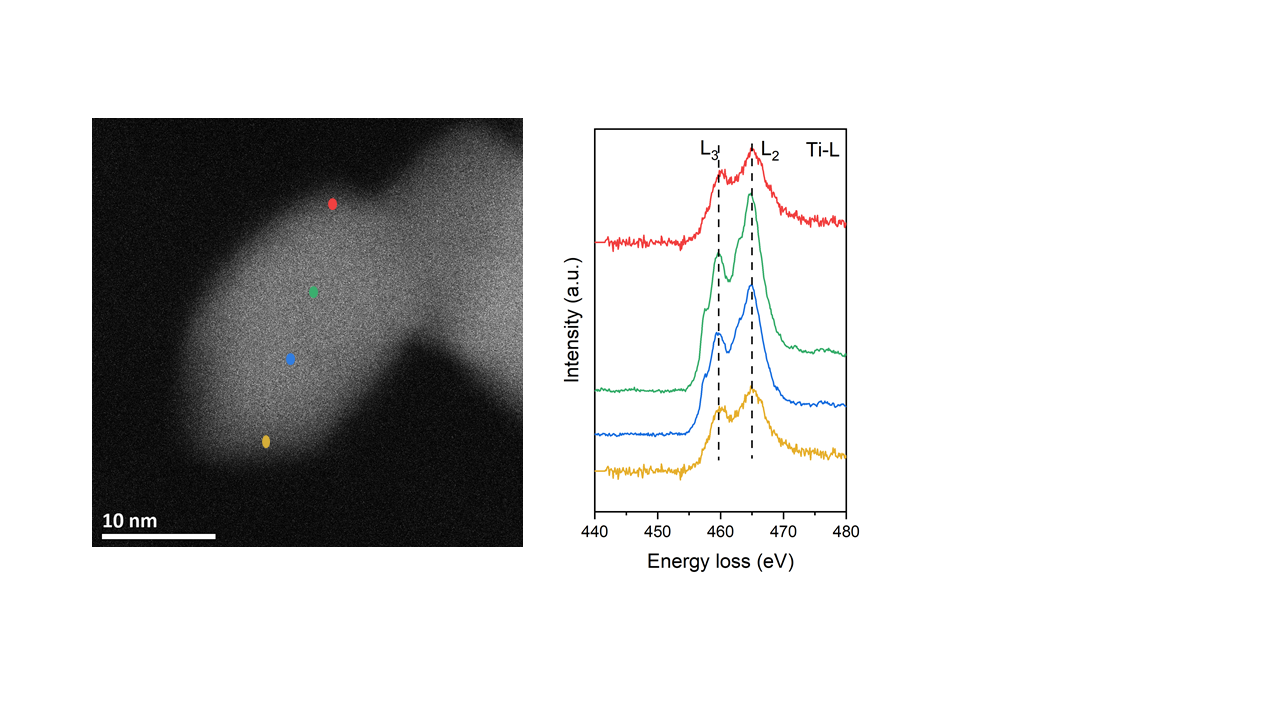


**Supplementary Fig. 6 |** HAADF-STEM image of c-TiO_2_ marked with the positions where EELS spectra were recorded. And EELS spectra of Ti-L_2,3_ in c-TiO_2_.


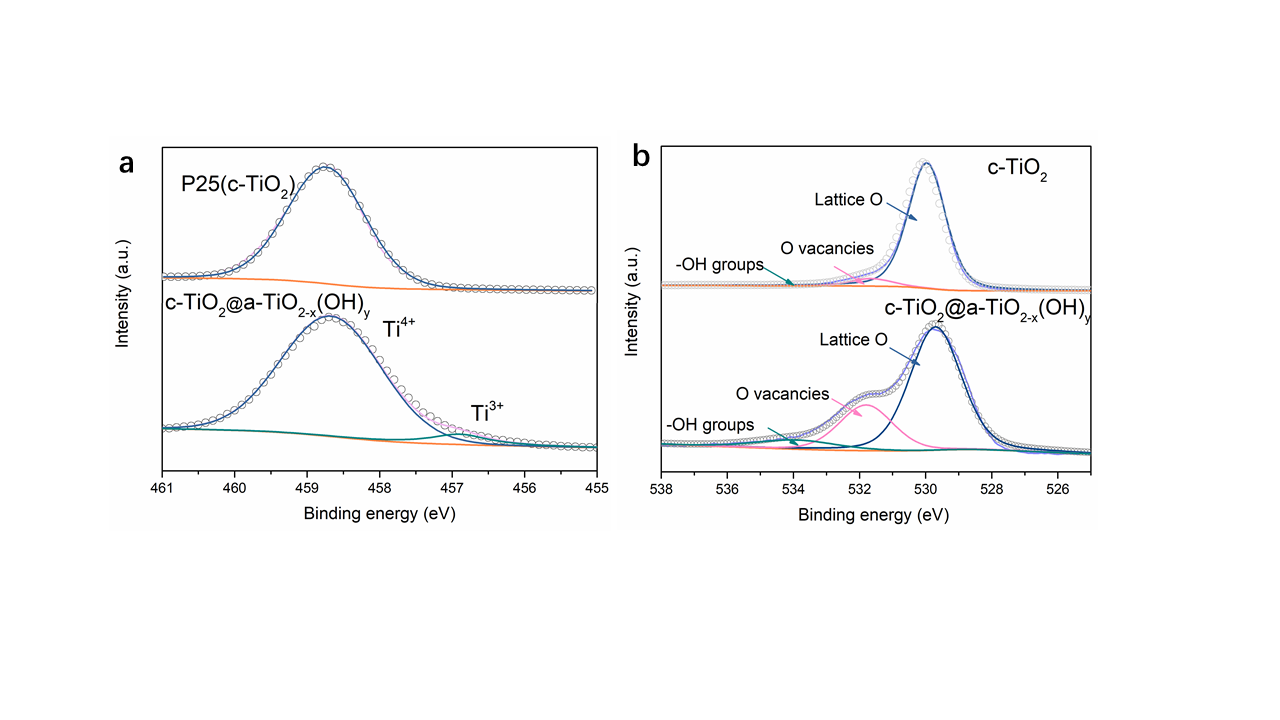


**Supplementary Fig. 7 |** **a.** Ti 2p and **b.** O 1s XPS spectra of c-TiO_2_@ a-TiO_2-x_(OH)_y_ and c-TiO_2_.


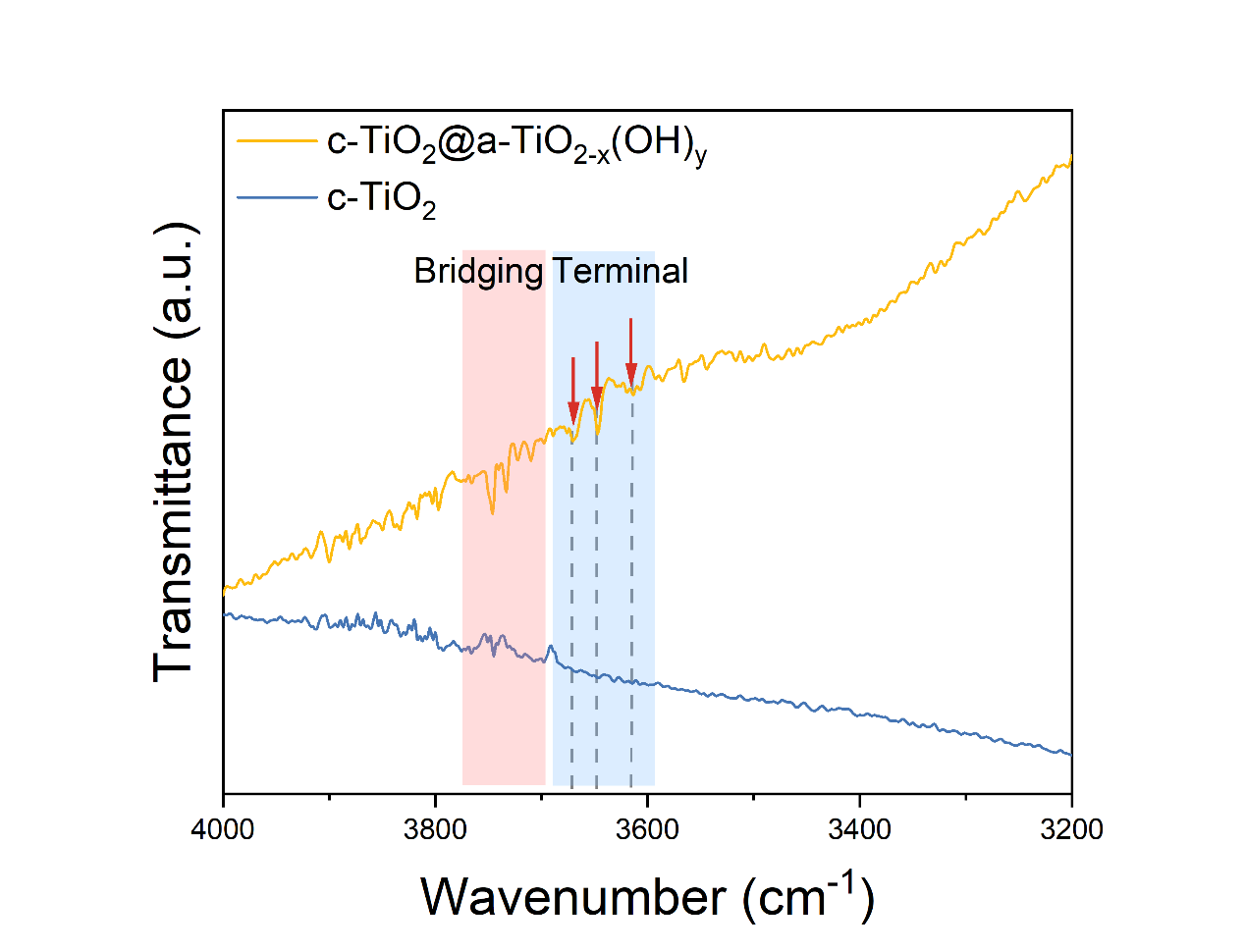


**Supplementary Fig. 8 |** ATR-FTIR spectra of c-TiO_2_@a-TiO_2-x_(OH)_y_ and c-TiO_2_.

According to previous infrared studies on titania surface, the stretching modes of terminal OH groups appear at frequency of 3610~3680 cm^-1^, while the bands at 3690~3750 cm^-1^ are assigned to bridging OH groups.[^1^](#_ENREF_1)^,^ [^2^](#_ENREF_2) As shown in Supplementary Fig. 7, weak peaks in bridging region emerged on the c-TiO_2_ sample, while large peak intensities with increased peak numbers were observed on the c-TiO_2_@ a-TiO_2-x_(OH)_y_ surface. Further, the notable new peaks at 3615, 3650 and 3674 cm^-1^ showed in c-TiO_2_@ a-TiO_2-x_(OH)_y_, hingting the increased population, heterogeneity of terminal and bridging OH on a-TiO_2-x_(OH)_y_ surface as compared to pristine c-TiO_2_. The corresponding bending peak at 1340 cm^-1^ also confirmed the formed teminal OH on the surface of c-TiO_2_@ a-TiO_2-x_(OH)_y_. This is reasonable given the disorder surface of self-trapped polarons and various Ti sites of heterogeneous coordinations, which electronically and/or geometrically intereact with the bonded OH to broaden its stretching frequencies.

**Supplementary Fig. 9 |** Tauc plots for c-TiO_2_@a-TiO_2-x_(OH)_y_ of different stoichiometry, of which the x and y values increased from c-TiO_2_, 1-c-TiO_2_@ a-TiO_2-x_(OH)_y_, 2-c-TiO_2_@ a-TiO_2-x_(OH)_y_, 3-c-TiO_2_@ a-TiO_2-x_(OH)_y_ to 4-c-TiO_2_@ a-TiO_2-x_(OH)_y_.


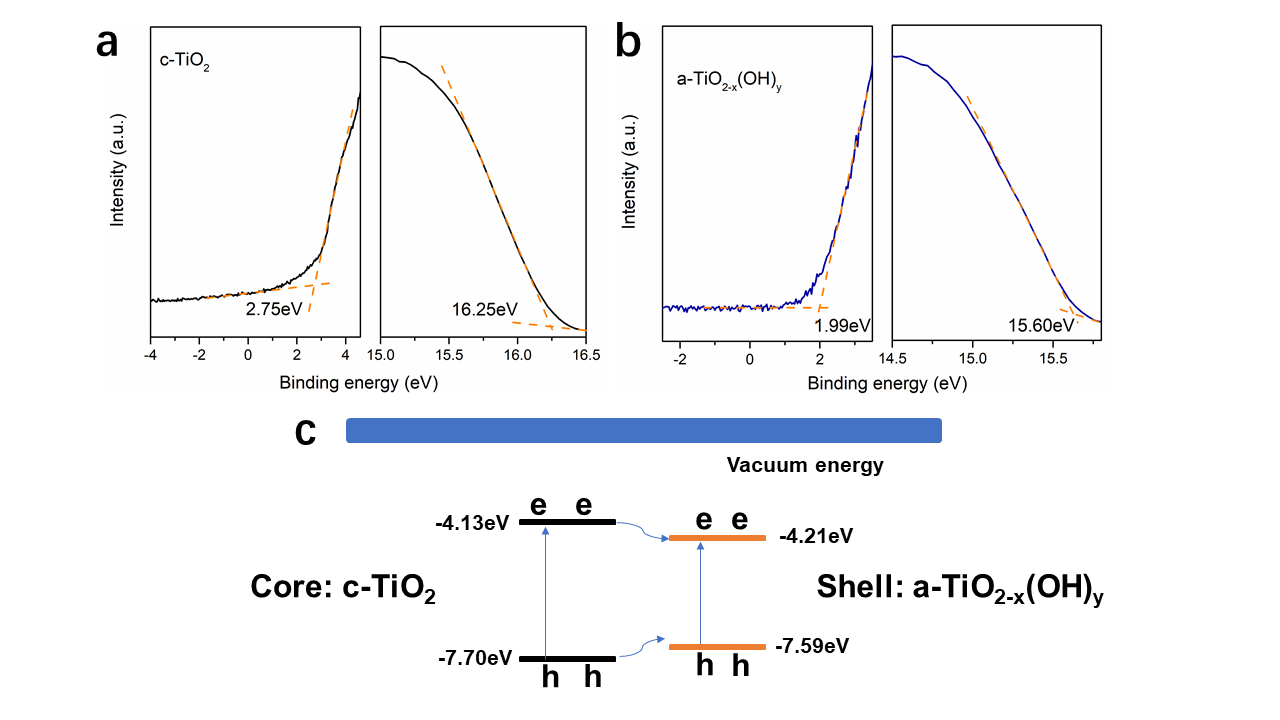


**Supplementary Fig. 10 |** **a,** **b.** UPS spectra of c-TiO_2_ (P25; a) and a-TiO_2-x_(OH)_y_ (b). **c.** Energy band alignment for the core@shell c-TiO_2_@a-TiO_2-x_(OH)_y_ with respect to the absolute vacuum energy scale (AVS).

The VB versus Fermi level and the end energy level (EEL) for c-TiO_2_ are determined to be *ca.* 2.75 eV and 16.25 eV by fitting a straight line to the leading edge, respectively. The work function (ψ) can be calculated to be ca. 4.95 eV according to the equation ψ= 21.2 -EEL. Thus, the the E_VB_ (vs. vacuum) is -7.70 eV and the E_CB_ (vs. vacuum) is -4.13 eV as determined from the band gap energy in the UV-vis DRS (3.57 eV). Accordingly, the E_VB_ (vs. vacuum) and the E_CB_ (vs vacuum) for a-TiO_2-x_(OH)_y_ are -7.59 eV and -4.21 eV, respectively.


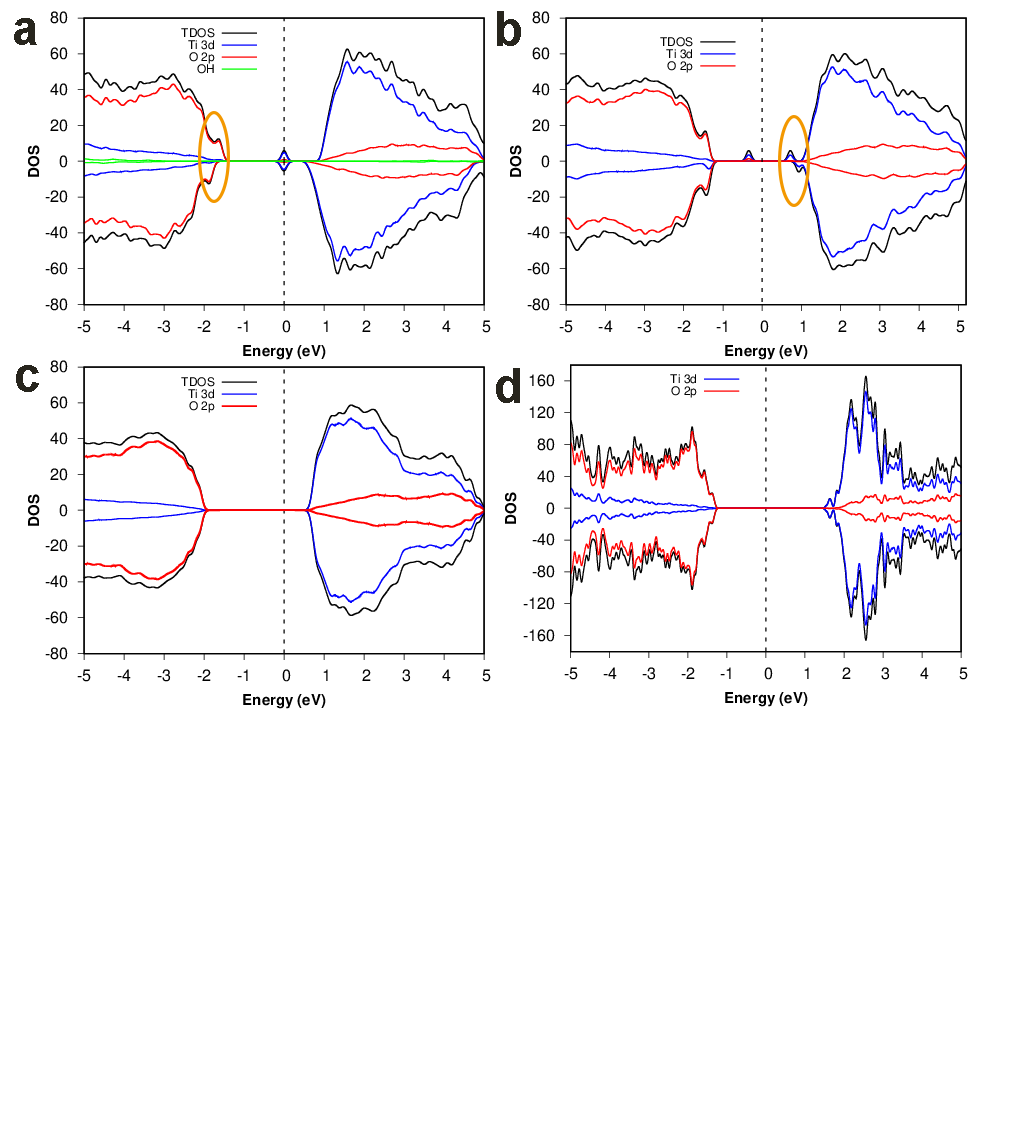


**Supplementary Fig. 11 |** The total density of states and projected density of states of the O p-, H s- and Ti d-orbitals of **a.** amorphous a-TiO_2-x_(OH)_y_, **b.** amorphous a-TiO_2-x_, **c.** amorphous a-TiO_2_, and **d.** crystalline c-TiO_2_ .





**Supplementary Fig. 12 |** Wavelength-dependent light absorption coefficient and penetration depth of the TiO_2_. The optical constants of anatase TiO_2_ are obtained from Ref[^3^](#_ENREF_3) by the average of parallel and perpendicular optical constants. The TiO_2_ is transparent to visible light and infrared light with energy smaller than its bandgap energy, and thus the penetration depth can only be calculated within the UV region.


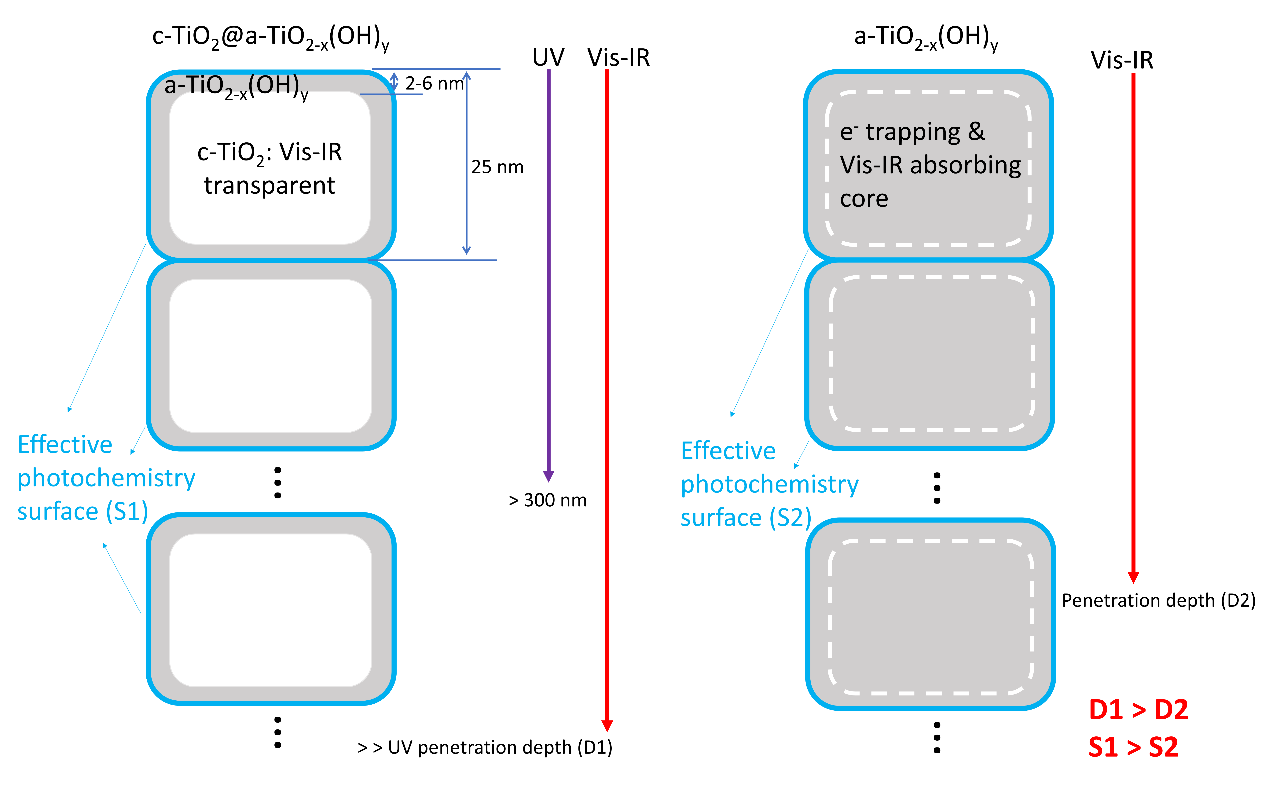


**Supplementary Fig. 13 |** Schematic of the light penetration depth and effective photochemistry surface area for c-TiO_2_@a-TiO_2-x_(OH)_y_ and pure a-TiO_2-x_(OH)_y_ in the stacked powder form. The total light absorption between the two samples should be the same which equals to (1-R), where R is the reflectance by the surface, but the Vis-IR light penetration depth of c-TiO_2_@a-TiO_2-x_(OH)_y_ should be larger than that of a-TiO_2-x_(OH)_y_ due to the Vis-IR transparent c-TiO_2_ core. Thus, the light accessible surface area of the former should be larger than the latter.

The penetration depth (D_p_) of UV light can be calculated for TiO_2_: The D_p_ = 1/α, where α is the light absorption coefficient that can be calculated using the formula of α= 4πk/λ, where k is the extinction coefficient that is well documented for white TiO_2_ [^3^](#_ENREF_3), and λ is the wavelength of the incident light. Based on above calculation, the wavelength-dependent D_p_ is listed above for white TiO_2_ (Fig. S11). According to the reflection spectra (Fig. 4a), c-TiO_2_ and a-TiO_2-x_(OH)_y_ demonstrates close UV-absorbing capacities, thus the similar penetration depth in UV region. Given the D_p_ is around 300 nm in UV region, the UV light will be partially absorbed by the 2─6 nm a-TiO_2-x_(OH)_y_ shell and then reach the core c-TiO_2_ in c-TiO_2_@a-TiO_2-x_(OH)_y_. Therefore, both the core c-TiO_2_ and shell a-TiO_2-x_(OH)_y_ will be excited by the UV light to yield photo electrons and holes. The 300 nm penetration depth also suggests the UV light can penetrate through ~12 stacked c-TiO_2_@a-TiO_2-x_(OH)_y_ particles (average size of 25 nm; Fig. S12).


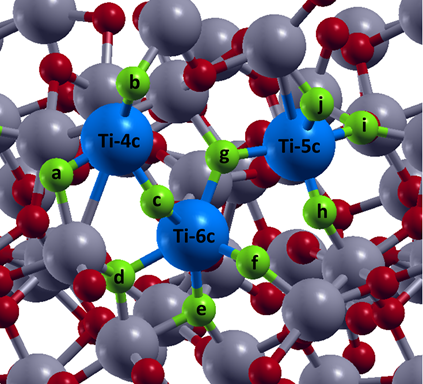


**Supplementary Fig. 14 |** The top view of an amorphous a-TiO_2_ surface indicating the 4-, 5-, and 6-coordinate Ti atoms (blue) with neighboring O atoms (green) that were removed one at a time to model amorphous TiO_2-x_ surface. The rest of the Ti and O atoms are represented in grey and red, respectively.


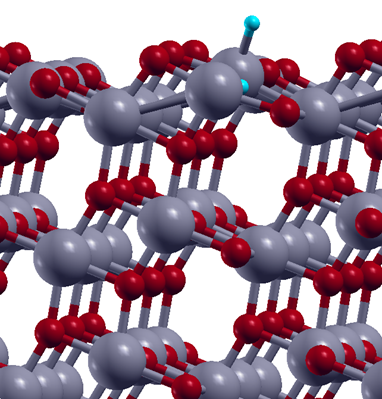


**Supplementary Fig. 15 |** Interaction of H_2_ on anatase (101) surface with oxygen vacancy (c-TiO_2-x_) at O3c site.


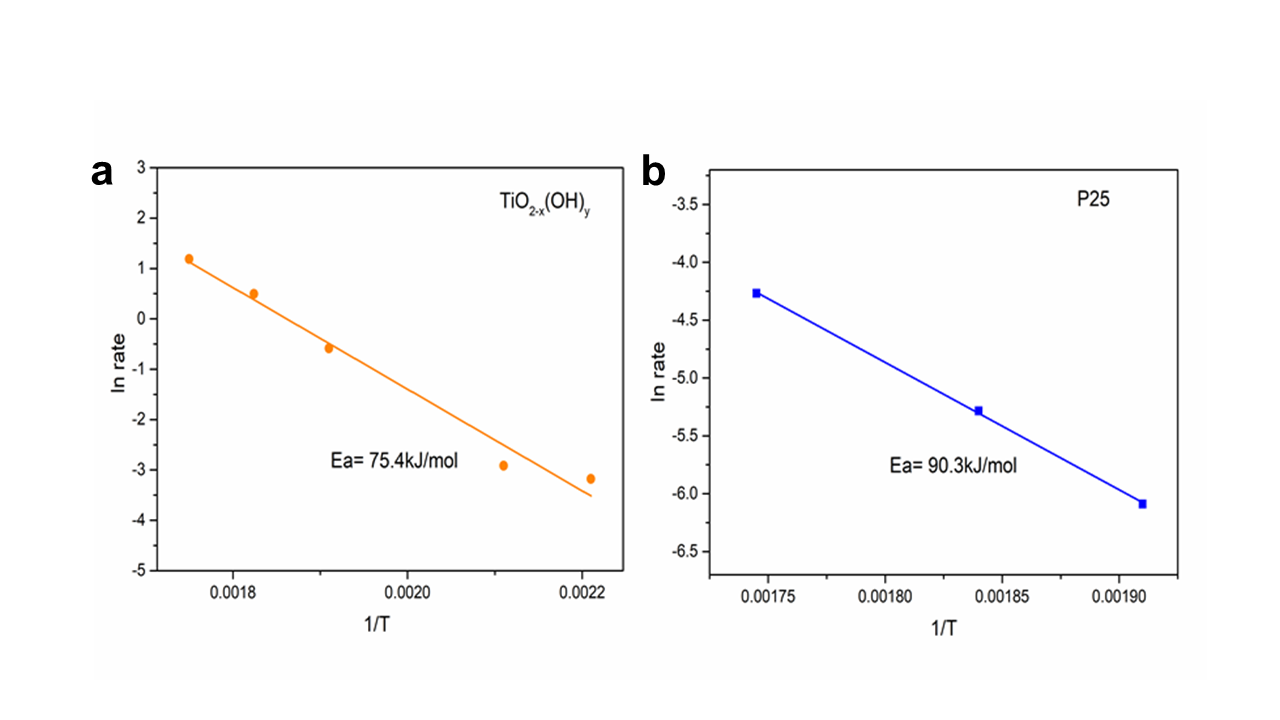


**Supplementary Fig. 16 |** The Arrhenius plots for the production rate of CO on **a.** c-TiO_2_@ a-TiO_2-x_(OH)_y_ and **b.** c-TiO_2_ recorded at different reaction temperatures in the flow reactor.





**Supplementary Fig. 17 |** Long-term stability of c-TiO_2_@ a-TiO_2-x_(OH)_y_ in a flow reactor, under both 0.8 W cm^−2^ light illumination and thermal activation, with 2 sccm of CO_2_ and 2 sccm of H_2_ gas flow, atmospheric pressure.

**Supplementary Fig. 18 |** XRD patterns of c-TiO_2_@a-TiO_2-x_(OH)_y_ before and after CO_2_ reduction.


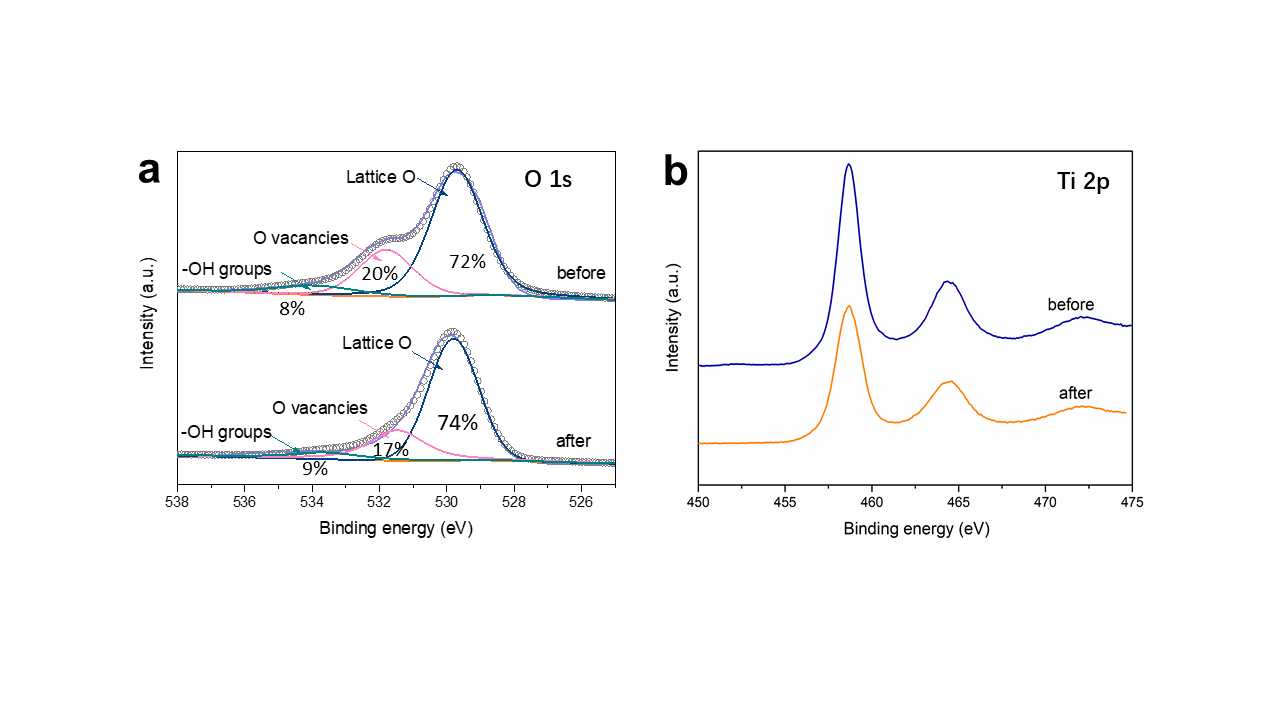


**Supplementary Fig. 19 |** **a.** O (1s) and **b.** Ti (2p) XPS spectra of c-TiO_2_@ a-TiO_2-x_(OH)_y_ before and after CO_2_ reduction. There is no significant change in O 1s and Ti 2p of XPS spectra between before and after reaction.

**Supplementary Fig. 20 |** GC-MS data from ^13^CO_2_ catalyst testing for c-TiO_2_@ a-TiO_2-x_(OH)_y_ using a CO_2_: H_2_ gas ratio of 1:1.





**Supplementary Fig. 21 |** CO production rates of of c-TiO_2_@ a-TiO_2-x_(OH)_y_, pristine P25 and 2% Na/P25 in a batch reactor under full-spectrum Xe light (4.0 W cm^−2^), CO_2_:H_2_=1:1 (15 psi CO_2_ and 15 psi H_2_).

**Supplementary Fig. 22 |** EPR spectra of c-TiO_2_@a-TiO_2-x_(OH)_y_.


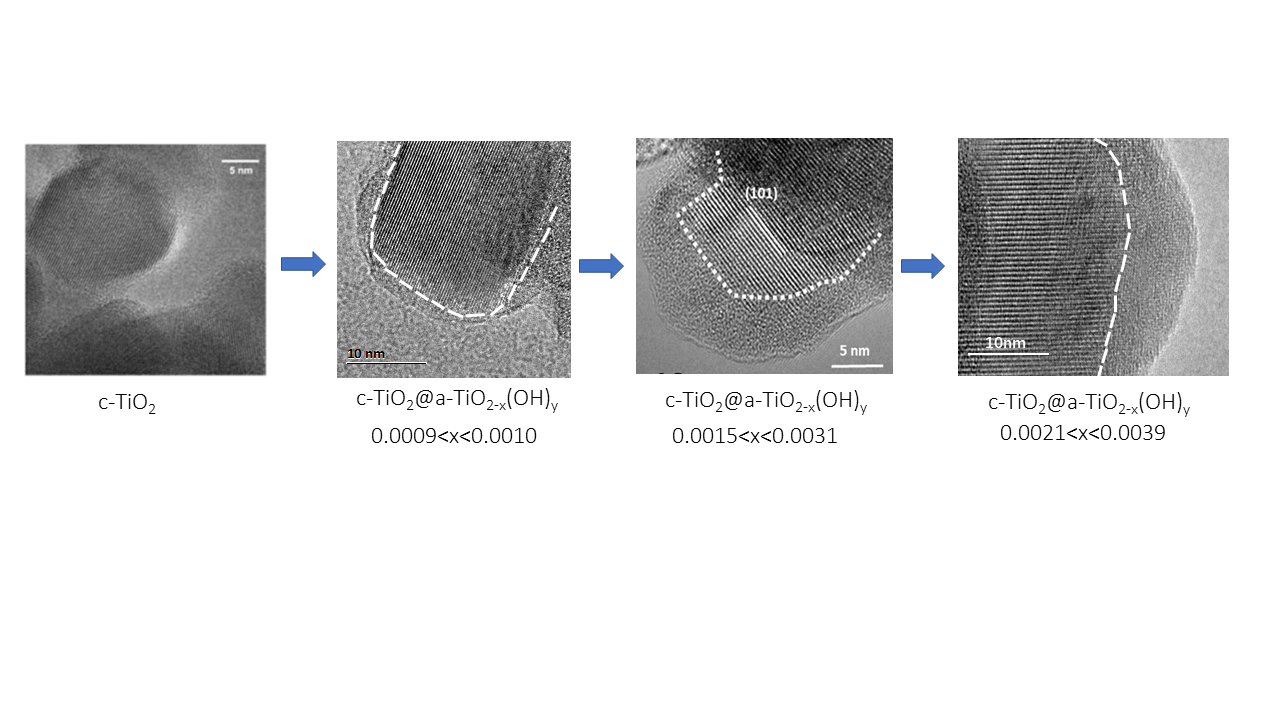


**Supplementary Fig. 23 |** HR-TEM micrograph of c-TiO_2_@a-TiO_2-x_(OH)_y_ with increasing stoichiometry. The amorphous/crystalline interfaces are marked with dotted lines.

**Supplementary Fig. 24 |** CO rate as a function of absorption wavelength for c-TiO_2_@ a-TiO_2-x_(OH)_y_ in an LED flow reactor, under both 0.83 W cm^−2^ single light illumination (UV, blue, green and red) and thermal activation (250 °C), with 2 sccm of CO_2_ and 2 sccm of H_2_ gas flow, atmospheric pressure.





**Supplementary Fig. 25 |** CO production rate of c-TiO_2_@ a-TiO_2-x_(OH)_y_ with different ratios of H_2_ and CO_2_ in a batch reactor, 4.0 W cm^-2^, CO_2_:H_2_=1:1 (15 psi CO_2_ and 15 psi H_2_), CO_2_:H_2_ = 3:1 (22.5 psi CO_2_ and 7.5 psi H_2_), CO_2_:H_2_=5:1 (25 psi CO_2_ and 5 psi H_2_).


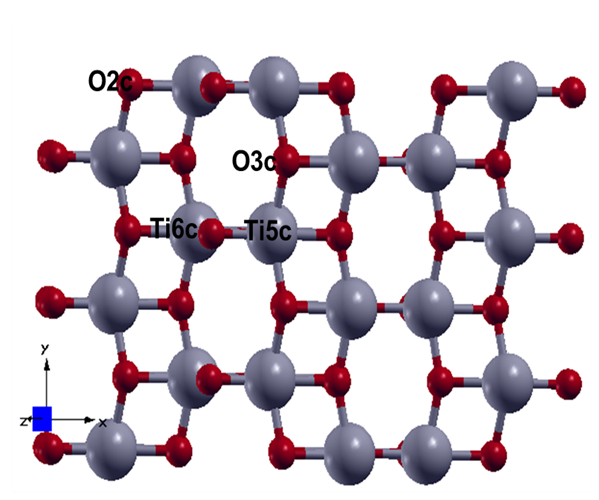


**Supplementary Fig. 26 |** The top view of the anatase (101) surface (c-TiO_2_) representing 2- and 3-coordinate O (red) as well as 5- and 6-coordinate Ti (grey) atoms.

**Supplementary Table 1**. PL-decay parameters for c-TiO_2_ and c-TiO_2_@ a-TiO_2-x_(OH)_y_.

| **Samples** | **τ_1_** | **A_1_** | **τ_2_** | **A_2_** | **τ_avg_** |
| --- | --- | --- | --- | --- | --- |
| **TiO_2_** | 2.65 | 674.3 | 14.2 | 253.3 | 10.4 |
| **c-TiO_2_@a-TiO_2-x_(OH)_y_** | 3.25 | 1048.9 | 51.7 | 48.9 | 23.6 |

The second order was fitted by using: $I\left( t \right)=A_{1}e^{(-t/\tau_{1})}+A_{2}e^{(-t/\tau_{2})},$ the averaged decay times (τ_avg_) are obtained from:

$\frac{{A_{1}\tau_{1}}^{2}+{A_{2}\tau_{2}}^{2}}{A_{1}\tau_{1}+A_{2}\tau_{2}}$ (1)

**Supplementary Table 2.** Formation energy of various a-TiO_2-x_ surfaces, formed by removing O atom from site ‘a’ to ‘j’, shown in Supplementary Fig. 9.

| **O site** | **a-TiO_2-x_ Formation Energy (Ry)** |
| --- | --- |
| **a** | 0.127871 |
| **b (least stable)** | 0.208071 |
| **c** | 0.070071 |
| **d** | 0.038071 |
| **e** | 0.030171 |
| **f** | 0.034471 |
| **g (most stable)** | -0.08133 |
| **h** | -0.06423 |
| **i** | -0.06643 |
| **J** | 0.048271 |

**Supplementary Table 3.** Photocatalytic performance of CO_2_ reduction over oxide-based catalysts.

| **Catalyst** | **Reactor** | | **Condition** | **Gas**  **composition** | **Production**  **rate (mmol⋅g^-1^⋅h^-1^)** | |
| --- | --- | --- | --- | --- | --- | --- |
| **1%Pd/TiO_2_** [**^4^**](#_ENREF_4) | | batch | 150 W Hg lamp | H_2_/CO_2_=4:1,  20 bar | | CH_4_: 0.3556,  CO: 0.0463  C_2_H_6_: 0.0396 |
| **3%N doped**  **TiO_2_** [**^5^**](#_ENREF_5) | | flow | 200 W Hg lamp,  100°C | H_2_/CO_2_=1:1,  1 bar | CO: 0.056  CH_4_: 0.0021 | |
| **3%Cu doped**  **TiO_2_** [**^5^**](#_ENREF_5) | | flow | 200 W Hg lamp,  100°C | H_2_/CO_2_=1:1,  1 bar | CO: 0.763  CH_4_: 0.0042 | |
| **0.5 % Au-10%MMT- TiO_2_** [**^6^**](#_ENREF_6) | | flow | 200 W Hg lamp,  100°C | H_2_/CO_2_=1:1,  1 bar | CO: 1.2 | |
| **TiO_2_/MOF (NH_2_-UiO-66)** [**^7^**](#_ENREF_7) | | Batch | 150 W Xe lamp | CO_2_/H_2_=1:1.5, 1.15 bar | CO: 0.0052 | |
| **0.03%Bi doped In_2_O_3-x_(OH)_y_** [**^8^**](#_ENREF_8) | | batch | 1000 W Blue metal halide bulb 150°C | H_2_/CO_2_=1:1,  2 bar | CO: 0.00132 | |
| **rh-In_2_O_3-x_(OH)_y_** [**^9^**](#_ENREF_9) | | flow | 130 W Xe lamp,  270°C | H_2_/CO_2_=3:1  1 bar | CO:1.03  CH_3_OH: 0.18 | |
| **c-TiO_2_@a-TiO_2-x_(OH)_y_ (This work)** | | batch | 300 W Xe lamp | H_2_/CO_2_=1:1  2 bar | CO: 5.3 | |
| **c-TiO_2_@a-TiO_2-x_(OH)_y_ (This work)** | | batch | 300 W Xe lamp | H_2_/CO_2_=1:5  2 bar | CO: 11.2 | |

**Supplementary Table 4.** Estimation of local temperature for c-TiO_2_@a-TiO_2-x_(OH)_y_ in the batch reactor system using ASPEN Plus, with the initial CO_2_/H_2_ ratio of 5:1 and irradiation intensity of 4.0 W‧cm^-2^. Upon reaching reaction equilibrium, the conversion of CO_2_ was 2.5%, corresponding to a CO concentration of 22,245 ppmv.

Wet basis mode (assuming product water reaches GC for detection):

| Temp (^o^C) | CO_2_ (ppmv) | CO (ppmv) | H_2_ (ppmv) | H_2_O (ppmv) | Sum (%) |
| --- | --- | --- | --- | --- | --- |
| 100 | 827,018 | 5,982 | 161,018 | 5,982 | 100 |
| 150 | 820,373 | 12,627 | 154,373 | 12,627 | 100 |
| 200 | 810,777 | 22,223 | 144,777 | 22,223 | 100 |
| 250 | 798,768 | 34,233 | 132,767 | 34,233 | 100 |
| 300 | 785,289 | 47,711 | 119,289 | 47,711 | 100 |
| 350 | 771,377 | 61,622 | 105,378 | 61,622 | 100 |
| 400 | 757,914 | 75,086 | 91,914 | 75,086 | 100 |
| 450 | 745,505 | 87,495 | 79,505 | 87,495 | 100 |
| 500 | 734,478 | 98,522 | 68,478 | 98,522 | 100 |
| 550 | 724,936 | 108,064 | 58,936 | 108,064 | 100 |
| 600 | 716,827 | 116,173 | 50,827 | 116,173 | 100 |
| 650 | 710,018 | 122,982 | 44,018 | 122,982 | 100 |
| 700 | 704,339 | 128,661 | 38,339 | 128,661 | 100 |

Dry basis mode (assuming the water condensed in the lines before reaching the GC detector)

| Temp (^o^C) | CO_2_ (ppmv) | CO (ppmv) | H_2_ (ppmv) | Sum (%) |
| --- | --- | --- | --- | --- |
| 100 | 831,995 | 6,018 | 161,987 | 100 |
| 150 | 830,864 | 12,788 | 156,347 | 100 |
| 200 | 829,204 | 22,728 | 148,068 | 100 |
| 250 | 827,081 | 35,446 | 137,473 | 100 |
| 300 | 824,633 | 50,101 | 125,266 | 100 |
| 350 | 822,033 | 65,669 | 112,298 | 100 |
| 400 | 819,443 | 81,182 | 99,376 | 100 |
| 450 | 816,987 | 95,884 | 87,128 | 100 |
| 500 | 814,749 | 109,289 | 75,962 | 100 |
| 550 | 812,767 | 121,157 | 66,076 | 100 |
| 600 | 811,049 | 131,443 | 57,508 | 100 |
| 650 | 809,582 | 140,227 | 50,190 | 100 |
| 700 | 808,341 | 147,659 | 44,000 | 100 |

**Supplementary Note**

Calculation of turnover frequency (TOF)

TOF = $\frac{number of produced molecues}{number of active sites total*reaction time}$ (2)

NA = 6.022 x 10 ^17^ μmol ^-1^

Number of produced molecules per hour = $\frac{CO rate * NA}{total reaction time}$ (3)

Total reaction time = 1 h

Number of surface O atoms at exposed facet = 3.07 x 10^19^ m^-2^ (4)

Pristine c-TiO_2_ rate: 15.1 μmol h^-1^g^-1^ → 0.34 μmol h^-1^ m^-2^ (accoring to the surface area: 45 m^2^ g^-1^ )

The number of produced molecules per hour for pristine c-TiO_2_ is 2.02 x 10^17^ m^-2^ h^-1^

c-TiO_2_@ a-TiO_2-x_(OH)_y_ rate: 5300 μmol h^-1^g^-1^ → 46.90 μmol h^-1^ m^-2^ (accoring to the surface area: 113 m^2^ g^-1^ )

The number of produced molecules per hour for c-TiO_2_@ a-TiO_2-x_(OH)_y_ is 2.82 x 10^19^ m^-2^ h^-1^

TOF (c-TiO_2_ )= 2.02 x 10^17^ m^-2^ h^-1^ / 3.07 x 10^19^ m^-2^=0.0066 h ^-1^  (5)

Assume all [O] will be used for reaction

Number of active sites = number of SFLPs =number of [O] = 3.07 x 10^19^ m^-2^ * [O] value obtained from EPR (0.0031/(2-0.0031)) = 4.76 x 10^16^ m^-2^ (6)

TOF (c-TiO_2_@ a-TiO_2-x_(OH)_y_ ) = 2.82 x 10^19^ m^-2^ h^-1^ / 4.76 x 10^16^ m^-2^ = 592 h ^-1^ (7)

The apparent quantum yield (AQY) was calculated according to the following equation.

$AQY=\frac{N(electrons)}{N(photons)}*100\%$ (8)

where N(electrons) and N(photons) represent the number of reacted electrons and the number of incident photons, respectively. According to the chemical equation (CO_2_ + H_2_ → CO + H_2_O), N(electrons) =  N(CO) = M(CO)N_A_, where N(CO), M(CO) and N_A_ represent the number of produced CO molecules, the number of moles of CO and Avogadro’s constant, respectively.

In this study，N(photon), is estimated from the light intensity dispersion of the Xe lamp (Supplementary Fig. 27) and the UV-vis-NIR absorption spectra.

$N_{photon}=\int_{300nm}^{2400nm} \frac{Light intensity*I\%*A\%*illmination area*time}{Average single photo energy*N_{A}}$ (9)

Where the light intensity is 4 W, illumination area is 1 cm^2^, I% is the percentage of the Xe light intensity at certain wavelength (Figure R4), A% is the light harvesting efficiency at certain wavelength according to the absorption spectra (Figure 1b in manuscript), time is 3600s. The average single photon energy (Ephoton) is figured out using the equation: Eλ = hc/λ, where h is the Planck constant, c indicates speed of light, and λ is the wavelength.

Thus, we can figure out that AQY for c-TiO_2_@a-TiO_2-x_(OH)_y_ is 0.09%.


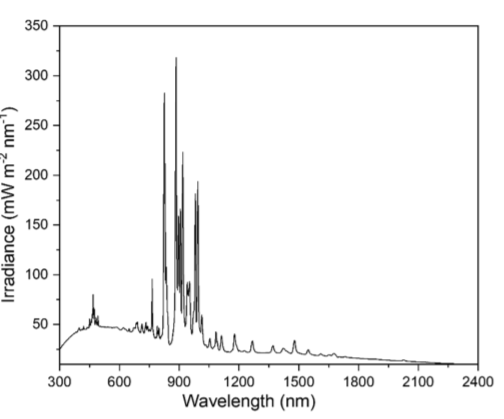


**Supplementary Fig. 27 |** The spectra of 300 W Xe lamp.

**Supplementary References**

1. Jackson P, Parfitt GD. Infra-red study of the surface properties of rutile. Water and surface hydroxyl species. *Trans. Faraday Soc.* 1971, **67**(0)**:** 2469-2483.

2. Liu C*, et al.* Structure–activity relationship of surface hydroxyl groups during NO_2_ adsorption and transformation on TiO_2_ nanoparticles. *Environ. Sci. Nano* 2017, **4**(12)**:** 2388-2394.

3. Palik ED. *Handbook of optical constants of solids*. Elsevier Science, 1998.

4. Li N*, et al.* Enhanced photocatalytic performance toward CO_2_ hydrogenation over nanosized TiO_2_-loaded Pd under UV irradiation. *J. Phys. Chem. C* 2017, **121**(5)**:** 2923-2932.

5. Tahir M, Tahir B. Dynamic photocatalytic reduction of CO_2_ to CO in a honeycomb monolith reactor loaded with Cu and N doped TiO_2_ nanocatalysts. *Appl. Surf. Sci.* 2016, **377:** 244-252.

6. Tahir M. Synergistic effect in MMT-dispersed Au/TiO_2_ monolithic nanocatalyst for plasmon-absorption and metallic interband transitions dynamic CO_2_ photo-reduction to CO. *Appl. Catal., B* 2017, **219:** 329-343.

7. Crake A, Christoforidis KC, Kafizas A, Zafeiratos S, Petit C. CO_2_ capture and photocatalytic reduction using bifunctional TiO_2_/MOF nanocomposites under UV–vis irradiation. *Appl. Catal., B* 2017, **210:** 131-140.

8. Dong Y*, et al.* Tailoring surface frustrated lewis pairs of In_2_O_3−x_(OH)_y_ for gas-phase heterogeneous photocatalytic reduction of CO_2_ by isomorphous substitution of In^3+^ with Bi^3+^. *Adv Sci* 2018, **5**(6)**:** 1700732.

9. Yan T*, et al.* Polymorph selection towards photocatalytic gaseous CO_2_ hydrogenation. *Nat. Commun.* 2019, **10**(1)**:** 2521.
