# Supplementary material for: Engineered disorder in CO2 photocatalysis
Source: Nat Commun. 2022 Nov 23;13:7205. doi: 10.1038/s41467-022-34798-1 (PMC9684568; doi:10.1038/s41467-022-34798-1)
Supplement: Supplementary file 3 — Description of Additional Supplementary Files [file 41467_2022_34798_MOESM3_ESM.pdf]

Description of Additional Supplementary Files

File Name: Supplementary Movie 1
Description: The video of HRTEM measurement and corresponding Fast Fourier transform pattern for the c-TiO_2@a-$TiO_{2-x}(OH)_y$.
